# Biomimetic mineralized hydrogels loaded with strontium-proanthocyanidins coordination particles for bone defect repair

**DOI:** 10.1093/rb/rbag185

**Published:** 2026-09-07

**Authors:** Fengkai Yang, Yijian Xie, Chengye Jiang, Xiang Jia, Xinshuo Liu, Lihua Li, Yifan Zhang, Mingyan Zhao, Changren Zhou

**Affiliations:** Stem Cell Research and Cellular Therapy Center, Affiliated Hospital of Guangdong Medical University, Zhanjiang 524001, China; Department of Materials Science and Engineering, Engineering Research Center of Artificial Organs and Materials, Jinan University, Guangzhou 510632, China; Department of Materials Science and Engineering, Engineering Research Center of Artificial Organs and Materials, Jinan University, Guangzhou 510632, China; Department of Materials Science and Engineering, Engineering Research Center of Artificial Organs and Materials, Jinan University, Guangzhou 510632, China; Department of Materials Science and Engineering, Engineering Research Center of Artificial Organs and Materials, Jinan University, Guangzhou 510632, China; Department of Materials Science and Engineering, Engineering Research Center of Artificial Organs and Materials, Jinan University, Guangzhou 510632, China; Institute of Dermatology and Venereal Diseases, Affiliated Hospital of Guangdong Medical University, Zhanjiang 524001, China; Department of Materials Science and Engineering, Engineering Research Center of Artificial Organs and Materials, Jinan University, Guangzhou 510632, China; Stem Cell Research and Cellular Therapy Center, Affiliated Hospital of Guangdong Medical University, Zhanjiang 524001, China; Department of Materials Science and Engineering, Engineering Research Center of Artificial Organs and Materials, Jinan University, Guangzhou 510632, China

**Keywords:** sodium alginate, strontium ion, proanthocyanidins, coordination, bone repair

## Abstract

Impaired vascularization and persistent inflammation strongly impede the healing of bone defects. To overcome these challenges, strontium-proanthocyanidins (PA) coordination particles (SrPA) with a particle size distribution of 600–900 nm were prepared. These SrPA particles were dispersed into sodium alginate (SA) solution pre-mixed with hydroxyapatite (HAp) and glutamate (Glu). A dual cross-linking process with HAp and calcium (Ca^2+^) increased the compressive strength of the resulting SrPA/HAp/glutamate/SA (nnGS) hydrogel by approximately 43-fold. As demonstrated by *in vitro* experiments, the release of PA from the nnGS modulated the inflammatory response and scavenged oxidative stress, whereas the release of strontium (Sr^2+^) promoted angiogenesis. Sr^2+^, PA and Ca^2+^ may contribute to osteogenic differentiation, collectively influencing the expression of osteogenesis-related genes. The signaling pathways associated with osteogenesis and inflammation were the most enriched. The results of *in vivo* experiments indicated that the nnGS hydrogel promoted angiogenesis, modulated the inflammatory microenvironment and accelerated bone repair at the defect site. By simultaneously addressing inflammation, vascularization and osteogenic differentiation, this study provides a novel strategy for treating bone defects.

## Graphical abstract

**Graphical Abstract:**
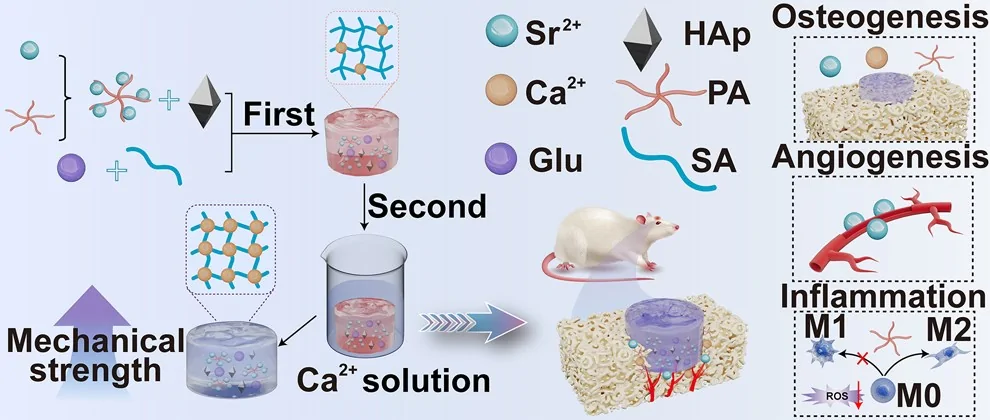

## Introduction

Bone defect repair remains a major challenge for orthopedic surgeons. Large bone defects resulting from high-energy trauma, tumor resection or infection often exceed the self-healing ability of bone tissue, leading to permanent functional impairment [1–3]. Although autologous bone transplantation remains the “gold standard,” its application is limited by donor site complications and secondary surgical trauma [4–6]. Bone injury is characterized by inflammation in the initial stage; however, excessive or chronic inflammation severely impedes tissue repair [7]. As bone is a highly vascularized tissue, its regeneration depends on an adequate blood supply [8]. Following a bone defect, a decrease in local blood perfusion causes tissue hypoxia, shifting cellular metabolism toward anaerobic pathways. Vascular disruption not only impairs the delivery of oxygen, nutrients and growth factors to osteogenic cells but also hinders the clearance of inflammatory metabolites and the recruitment of anti-inflammatory M2 macrophages to the site of injury [9, 10]. Thus, regulation of inflammation and vascularization collectively shapes the osteogenic microenvironment.

Metal ions, such as calcium (Ca^2+^), strontium (Sr^2+^), zinc, magnesium, copper and iron, play indispensable roles in bone regeneration [11, 12]. They regulate osteogenic signaling pathways and affect the activity of osteoblasts and osteoclasts [13]. Trace amounts of metal ions can also promote angiogenesis, modulate the immune microenvironment and regulate neural and metabolic functions [14]. The effects of most metal ions are concentration dependent; specifically, low to moderate concentrations promote bone regeneration, whereas exceeding a threshold may have cytotoxic or inhibitory effects. For example, Ca^2+^ promotes the proliferation of osteoblasts at low concentrations and enhances differentiation and matrix mineralization at moderate concentrations but becomes cytotoxic at high concentration [15]. At low concentrations, Sr^2+^ functions through a “dual-regulatory mechanism,” that promotes the nuclear translocation of β-catenin by activating the Wnt/β-catenin pathway and upregulating the expression of RUNX2 to drive osteogenic differentiation of mesenchymal stem cells [16–18]. Moreover, they inhibit RANKL-mediated activation of osteoclasts, thereby increasing bone mineral density (BMD) and the mechanical strength of newly formed bone [19–21]. Therefore, the concentration of metal ions affects the therapeutic effect. To circumvent this problem, the release of metal ions significantly affects the therapeutic outcome, and their release should be tightly regulated.

Plant polyphenols such as proanthocyanidins (PA) provide new opportunities for reducing oxidative stress at bone defect sites due to their antioxidative, anti-inflammatory and pro-angiogenic effects [22–25]. The polyphenolic hydroxyl groups in PA contain oxygen atoms with lone pair electrons, enabling them to act as electron donors and form stable coordination complexes with metal ions possessing empty orbitals [26, 27]. The coordination network increases the loading stability of active components within the material system and improves the structural integrity and mechanical stability through multidentate coordination. When the local wound microenvironment changes, particularly under conditions of low pH and high reactive oxygen species (ROS) levels, the coordination bonds between metal ions and polyphenols undergo dynamic changes. This leads to the gradual dissociation of the complex structure and facilitates the release of PA and metal ions [28, 29]. Unlike the rapid diffusion-driven release caused by physical encapsulation, this dynamic coordination mechanism can slow the leakage of active components, decrease the risk of initial burst release and facilitate a milder and more sustained release of polyphenols and metal ions into the wound microenvironment [30, 31].

Many researchers have focused on improving the osteogenic microenvironment by developing and implementing sustained and effective treatment strategies [32–34]. Bone tissue engineering scaffolds, which can serve as bone graft alternatives, are designed to mimic and replace native bone tissue [35, 36]. These scaffolds are typically composed of highly biocompatible materials and enable the sustained release of active substances to promote new bone formation [37, 38]. However, several challenges remain, such as the need to more accurately replicate the complex structure of natural bone and develop materials with excellent bone-mimetic properties. Future studies should focus more on the development of multifunctional and intelligent scaffolds capable of concurrently supporting osteogenic, angiogenic and anti-inflammatory functions.

Based on this scientific rationale, in this study, a SrPA/HAp/glutamate (Glu)/SA (nnGS) composite hydrogel was developed using natural alginate polysaccharide as the matrix. Glu was blended with SrPA particles and HAp. The Ca^2+^ released from HAp rapidly cross-link with alginate to form a hydrogel. Immersing the hydrogel in a Ca^2+^ solution significantly improved its mechanical properties. The particles degraded and released Sr^2+^ and PA in the acidic environment at the defect site, which helped modulate the inflammatory microenvironment and promoted vascularization. This resulted in a combined effect involving Ca^2+^, Sr^2+^ and PA, providing a favorable regenerative microenvironment for bone repair. Animal experiments were conducted using a calvarial defect model in which the nnGS hydrogel promoted new bone regeneration, and its anti-inflammatory and pro-angiogenic effects were significantly stronger than those of the other treatments. This innovative strategy provides a theoretical foundation for developing bone repair materials that target inflammation and vascularization (Supplementary Figure S1).

## Materials and methods

### Materials

All chemical reagents, including anhydrous calcium chloride (99%), strontium chloride, PA, hydroxyapatite (HAp), sodium alginate (SA), glutamate (Glu), Ca^2+^ standard solution and Sr^2+^ standard solution, were purchased from Macklin (China). Cell culture reagents, including high-glucose DMEM, penicillin–streptomycin and phosphate-buffered saline (PBS; pH 7.4 and 6.5), were purchased from Gibco (USA). The CCK-8 assay kit was purchased from DOJINDO (Japan). A BCIP/NBT alkaline phosphatase colorimetric kit, a BCA protein assay kit and a live/dead cell staining kit were purchased from Beyotime Biotechnology (China). The ROS inducer was purchased from Bestbio (China). The DAPI staining solution was purchased from Acmec (China). The alkaline phosphatase (ALP) test kit was purchased from Nanjing Jiancheng Bioengineering Institute (China). Osteogenic differentiation basal medium and Alizarin Red S staining solution were purchased from Oricell (USA). SYBR Green dye and 4% paraformaldehyde (PFA) universal tissue fixative was purchased from Biosharp (China).

### Preparation and characterization of SrPA particles

#### Synthesis of SrPA particles

Briefly, 200 mg of strontium chloride and 100 mg of PA were separately dissolved in 200 mL of deionized water at room temperature. After ultrasonic-assisted dissolution (100 W, 10 min), the two solutions were mixed in a 500 mL light-protected reaction flask. The mixture was allowed to react for 24 h in the dark with continuous magnetic stirring at 500 rpm. After the reaction, the product was centrifuged at 10 000 rpm for 15 min to collect the precipitate, which was then washed 3–5 times with deionized water. Dark brown SrPA powder was obtained after vacuum drying at 50°C for 24 h and stored under dry and dark conditions.

#### Characterization of SrPA particles

The synthesized SrPA particles were characterized using several analytical techniques. X-ray diffraction (XRD; Rigaku Company, Miniflex 600, Japan) analysis was performed in the 2*θ* range of 0°–90° at a scanning rate of 5°/min. The particle size distribution was determined using a Mastersizer 2000 instrument (Malvern, UK). The chemical composition and elemental states were characterized using X-ray photoelectron spectroscopy (XPS; Thermo Scientific, K-Alpha+, USA). The morphological characteristics were examined, and elemental mapping was performed using a transmission electron microscope (TEM; FEI Tecnai, F20, USA) equipped with an energy-dispersive X-ray spectroscopy (EDS) detector.

#### Cytocompatibility of Sr^2+^, PA and SrPA particles

The cytotoxicity of Sr^2+^, PA, and SrPA particles was evaluated using Bone marrow mesenchymal stem cells (BMSCs, Oricell, HUXMA-01001). BMSCs were seeded in 24-well plates at a density of 5 × 10^4^ cells/well. SrPA particles were suspended in medium to prepare a series of concentrations (12.5, 25, 50, 75, 100, 125, 625 and 1250 μg/mL). The cells were treated with these media for 24 h. Cell viability was then evaluated by CCK-8 assay combined with live/dead staining to identify the optimal SrPA concentration with the best cytocompatibility. Next, the BMSCs were cultured with the optimized concentration of SrPA and with equivalent concentrations of Sr^2+^ (5.3 μg/mL SrCl_2_) and PA (10 μg/mL) alone for 1, 3 and 5 days. At each time point, cell viability was quantitatively evaluated using the CCK-8 assay, whereas cell survival was assessed by live/dead staining in parallel samples. Fluorescence images were acquired using an inverted fluorescence microscope (Zeiss Axio Vert. A1, Germany).

#### Evaluation of intracellular ROS scavenging ability

The regulatory effects of PA and SrPA on ROS-induced oxidative stress in BMSCs were evaluated using a DCFH-DA fluorescence probe (Beyotime). Four groups were included: a negative control (NC; unstimulated), a positive control (PC; ROS inducer only), a PA-treated group and a SrPA-treated group (both with ROS inducer stimulation). BMSCs were seeded in 24-well plates at a density of 1 × 10^5^ cells/well. At 70–80% confluence, cells in the PC, PA and SrPA groups were exposed to the ROS inducer at 50 μg/mL (diluted 1:1000 in culture medium) for 20 min to induce oxidative stress. After induction, the medium was removed, and the cells were washed with PBS. Fresh medium was then added according to the group assignment: normal medium for the NC and PC groups, medium containing 10 μg/mL PA for the PA group and medium containing 12.5 μg/mL SrPA for the SrPA group. Following a 12-h incubation, the medium was replaced with serum-free medium containing 10 μM DCFH-DA and incubated at 37°C in the dark for 30 min. The cells were then stained with DAPI for 5 min to label nuclei. After additional PBS washes, fluorescence images were captured using an inverted fluorescence microscope, and the fluorescence intensity was quantified using ImageJ software (version 1.53t; National Institutes of Health, Bethesda, MD, USA).

#### Evaluation of HUVECs migration

Human umbilical vein endothelial cells (HUVECs, CL-0675) were purchased from Procell (China). The effect of SrPA on the migration of HUVECs was quantitatively evaluated by scratch wound healing assay. HUVECs were seeded in 6-well plates at a density of 5 × 10^4^ cells/well and cultured at 37°C under 5% CO_2_ and 95% humidity until a confluent monolayer (>95%) formed. A uniform scratch was made in the monolayer using a sterile 200 μL pipette tip. Normal culture medium was used for the control group, and culture media containing Sr^2+^, PA and SrPA were prepared and added to the wells. Images of the wound areas were taken at fixed locations immediately after scratching (0 h) and at 12, 24 and 48 h post-scratching under an inverted microscope (Olympus BX53, Japan). The images were analyzed using ImageJ software to quantify the change in the scratch area. The cell migration rate (wound healing rate) was calculated using Equation (1)


(1) $$\text{Cell}\;\text{migration}\;\text{rate}\;(\%)=\left(S_{0}-S_{t}\right)/S_{0}\times 100\%$$

Here, *S*_0_ indicates the initial scratch area (0 h) and *S_t_* indicates the scratch area at a specific time point (12 h, 24 h or 48 h).

In the tube formation assay, 50 μL of Matrigel (Macklin) was evenly coated onto the bottom of a 24-well plate and left undisturbed at 37°C for 1 h. Subsequently, HUVECs were seeded onto the Matrigel coating at a density of 5 × 10^4^ cells/well. After seeding, the cells were cultured in media supplemented with Sr^2+^, PA and SrPA. Afterward, the treated cells were incubated at 37°C and 5% CO_2_ for 6 h. Images of the tubular structures formed by the cells were captured using a microscope, and the tubular network was quantitatively analyzed using ImageJ software.

### Preparation and characterization of nGS and nnGS hydrogels

The details of the preparation and characterization of the HAp/Glu/SA (nGS) and nnGS hydrogels are described in the Supporting Information.

### *In vivo* implantation

#### Rat skull defect model

All animal experiments were conducted following the National Research Council’s Guide for the Care and Use of Laboratory Animals and approved by the Animal Ethics Committee of the Affiliated Hospital of Guangdong Medical University (Approval No. AHGDMU-LAC-B-202406-0040).

Male Sprague–Dawley rats (6–8 weeks old, 200 ± 20 g) were obtained from SPF (Beijing) Biotechnology Co., Ltd. (License No. SYXK2022-0286). The animal experiment followed a completely randomized design, with rats allocated to four groups (Control, nnGS, nGS and HAp) according to a computer-generated random sequence before surgery. A total of six rats were used per group, with three rats sacrificed at 6 weeks and the other three at 12 weeks. After the rats were anesthetized with isoflurane (Orbiepharm, China), the cranial hair was removed and the scalp was incised under sterile conditions to expose the skull. Two full-thickness critical-size defects (5 mm in diameter) were created on the skull of each rat. Both defects in the same rat received the same treatment according to the pre-defined grouping, and the defect sites were either left untreated (control group) or implanted with the corresponding materials. Because the materials differed visibly in appearance, the surgeon performing the implantation could not be blinded to the material type. Afterward, the scalp was sutured closed. At weeks 6 and 12 post-surgery, the rats were euthanized. Calvarial samples were collected, fixed in 4% PFA for 24 h and subjected to micro-CT analysis for evaluation of bone regeneration. Major organs (heart, liver, spleen, lung and kidney) were also collected at 12 weeks for histological examination. Subsequent micro-CT region of interest (ROI) definition and quantitative analysis, histological image selection, immunofluorescence quantification and statistical analysis were performed by investigators blinded to group allocation.

#### Micro-CT scanning

The fixed calvarial specimens were scanned using a micro-CT system (MCT-Sharp, ZKKS, China) at 70 kV and 100 μA with a voxel size of 20 μm. Three-dimensional (3D) images were reconstructed, and bone mineral density (BMD) and bone volume/total volume (BV/TV) were calculated for quantitative assessment of new bone formation within the defect regions.

#### Histological analysis

After micro-CT scanning, the calvarial samples were decalcified, dehydrated and embedded in paraffin. The embedded tissues were sectioned into slices (5–10 μm thick) using a microtome. These sections were subjected to hematoxylin-eosin (H&E, G1120, Solarbio, China) and Masson’s trichrome staining (G1340, Solarbio), respectively. Major organs collected at 12 weeks were also processed for H&E staining to evaluate potential systemic toxicity of the implanted materials. All stained sections were observed and imaged under an inverted microscope.

#### Immunofluorescence staining

At 6 and 12 weeks post-implantation, paraffin sections of the calvarial defects were processed for immunofluorescence staining of RUNX2 (1:200, 660207, Zenbio, China), ALP (1:200, R381009, Zenbio), osteocalcin (OCN) (1:300, 23418-1-AP, Proteintech, China), CD86 (1:300, 30691-1-AP, Proteintech), CD206 (1:300, 18704-1-AP, Proteintech) and CD31 (1:300, 28083-1-AP, Proteintech). After deparaffinization and rehydration of the paraffin sections, antigen retrieval and permeabilization were performed. Afterward, the sections were blocked with BSA (A8020, Solarbio), followed by incubation with primary and secondary antibodies (1:300, A11034, Thermo Fisher Scientific, USA). Finally, the nuclei were counterstained with DAPI, and the slides were mounted. For quantitative analysis, images were acquired under identical microscope settings. Regions of interest were selected within the original calvarial defect area. For each animal, three nonconsecutive sections were analyzed, and the same anatomical region within the original defect area was used for quantification. The fluorescence intensity was quantified using ImageJ after background subtraction and normalized to the corresponding tissue area. The average value of three sections of each animal was used as one independent biological replicate for statistical analysis.

### Statistical analysis

All experiments were performed with at least three independent replicates. For *in vitro* assays, biological replicates were independently prepared from cell cultures or material samples, whereas for *in vivo* analyses, each animal was considered one biological replicate. The data are expressed as the mean ± standard deviation. Statistical analysis was conducted using SPSS. Data normality and homogeneity of variance were assessed using the Shapiro–Wilk test and Levene’s test, respectively. For comparisons among multiple groups at a single time point, one-way ANOVA was performed. When experiments included multiple groups and multiple time points, statistical comparisons among groups were conducted separately at each individual time point using one-way ANOVA. *Post hoc* multiple comparisons were performed using Tukey’s test for pairwise comparisons among groups or Dunnett’s test when comparisons were made against the control group. Multiple comparisons were controlled using the adjusted *P*-values generated by the corresponding *post hoc* tests. Differences were considered statistically significant at *P* < 0.05, with the following levels indicated: *P < 0.05, **P < 0.01 and ***P < 0.001; differences that did not meet this threshold (*P* ≥ 0.05) were considered not significant (ns).

## Results

### Characterization of SrPA particles

A series of physicochemical characterizations was performed to confirm the successful synthesis of SrPA particles. XRD analysis revealed a significant weakening and broadening of the characteristic diffraction peak of strontium chloride in the SrPA pattern, along with the emergence of amorphous dispersion peaks between 15° and 30° (Figure 1A). These features indicated a structurally disordered metal–organic complex formed between Sr^2+^ and the phenolic hydroxyl groups of PA. XPS analysis provided further evidence (Figure 1B–E). In the O1s spectrum, the dominant peak located at 532 eV corresponded to Sr–O bonding, confirming the presence of Sr^2+^ coordinated with oxygen from PA. Additionally, compared with Sr^2+^ in strontium chloride, the doublet peaks in the Sr3d spectrum observed at 135.0 eV (Sr3d_3/2_) and 133.0 eV (Sr3d_5/2_) showed a binding energy decrease of 1.5 eV, accompanied by significant peak broadening.

**Figure 1:**
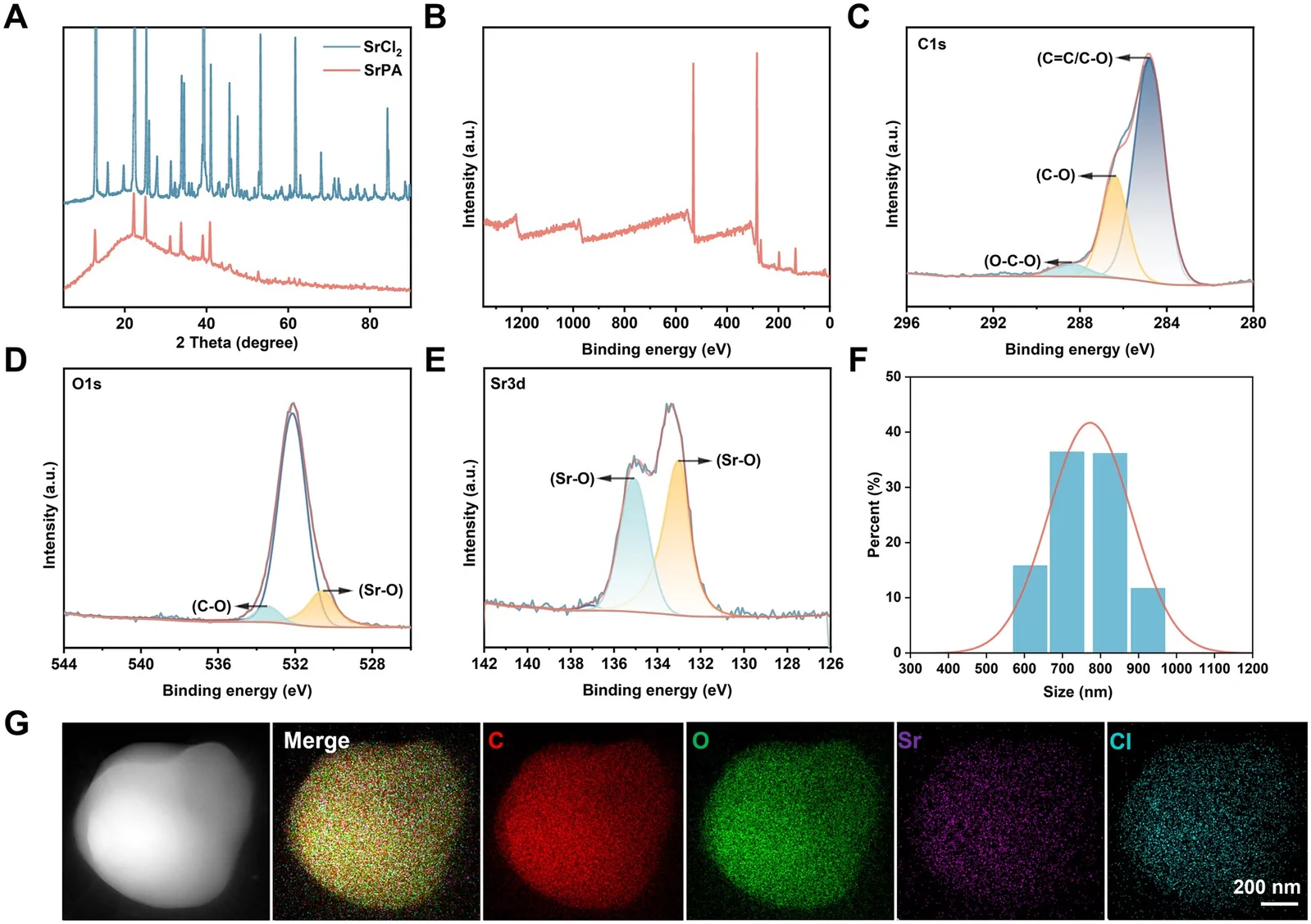
(**A**) XRD patterns of strontium chloride and SrPA; (**B**) Full-XPS spectrum of SrPA; (**C–E**) Fine-XPS spectra of the C1s, O1s and Sr3d regions of SrPA; (**F**) Histogram of the particle size distribution of SrPA; (**G**) TEM elemental mapping of SrPA.

These shifts indicated that a coordinated interaction between Sr^2+^ and PA in the SrPA complex. Particle size analysis showed that the SrPA particles were primarily concentrated in the range of 600–900 nm with a relatively uniform distribution (Figure 1F). TEM images revealed that the particles had an irregular spherical morphology and an average particle size of approximately 700 nm. Elemental mapping demonstrated a highly uniform co-distribution of C, O and Sr within the particles, with trace amounts of chlorine residues also detected (Figure 1G).

To determine a suitable concentration, BMSCs were co-cultured with different concentrations of SrPA (12.5–1250 μg/mL) for 24 h. The interaction between the particles and cells significantly increased in a dose-dependent manner (Supplementary Figure S2). A SrPA concentration of 12.5 μg/mL was selected for subsequent experiments, as it exhibited good cytocompatibility (cell viability >100%) and continuously promoted cell proliferation. Subsequent experiments were conducted on the nnGS hydrogel loaded with 12.5 μg/mL SrPA particles.

### Evaluation of cellular activity of SrPA

In the cellular microenvironment, SrPA dissociates into Sr^2+^ and PA, and both components can influence cellular activity. BMSCs were co-cultured with 12.5 μg/mL SrPA particles for 1, 3 or 5 days. Live/dead cell staining showed that SrPA treatment did not alter the morphology of BMSCs, but an increase in cell proliferation was observed. Treatment with Sr^2+^ also promoted proliferation. The results of the CCK-8 assay on day 5 revealed that compared with the control group, both the Sr^2+^ and SrPA groups demonstrated significant proliferative effects, whereas the PA group did not significantly differ (Figure 2A and C). These findings indicate that 12.5 μg/mL SrPA promotes the proliferation of BMSCs.

**Figure 2:**
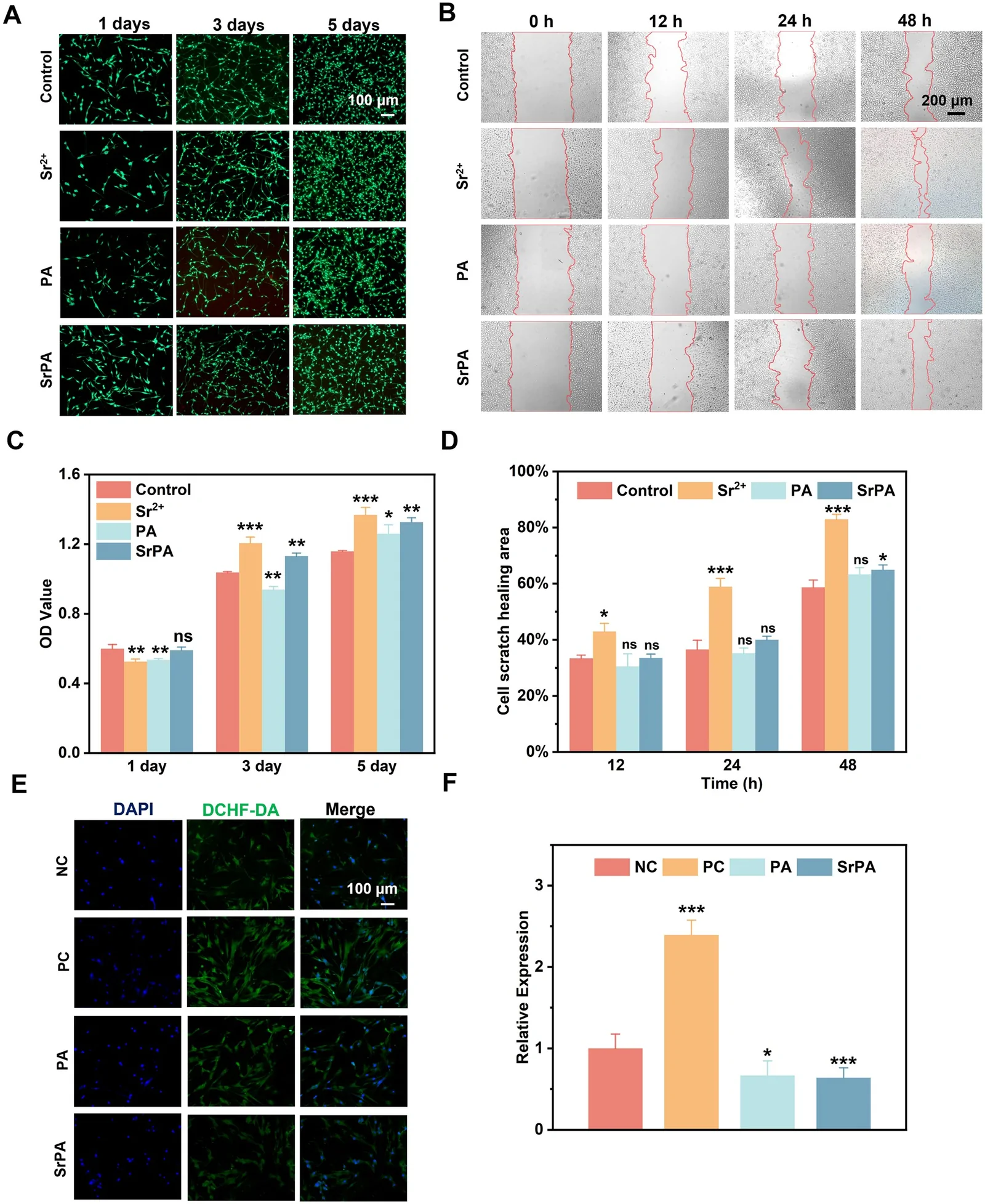
(**A**) Live –dead cell staining and (**C**) the viability of BMSCs treated with Sr^2+^, PA and SrPA for 1, 3 and 5 days; (**B**) Migration images and (**D**) quantitative migration rates of HUVECs cultured with Sr^2+^, PA and SrPA at 0, 12, 24 and 48 h; (**E**) Fluorescence images and (**F**) relative fluorescence intensity of ROS in BMSCs. (ns: not significant; *P < 0.05, **P < 0.01 and ***P < 0.001 vs. the control group; *n* = 3; *P*-values were calculated using one-way ANOVA with Dunnett’s multiple comparisons test).

The effect of SrPA on endothelial cell migration was evaluated by scratch assay (Figure 2B and D). Free Sr^2+^ exhibited significant pro-migration effects by 12 h, with effects intensifying over time. The scratch healing rate increased by 62.5% at 24 h and 85.3% at 48 h. In contrast, SrPA particles showed delayed pro-migratory effects, probably due to particle stability under conventional culture conditions (pH 7.4). SrPA-induced migration became evident after 24 h, reaching 68.2% at 48 h. The tube formation assay revealed that after 6 h of cultivation in different media, the Sr^2+^ group had the greatest tube length and the greatest number of nodes and junctions; compared with the control group, the SrPA group also showed significant improvements. These results indicate that Sr^2+^ supports angiogenic responses (Supplementary Figure S3).

At bone defect sites, infection and inflammation drive ROS accumulation, which impedes tissue repair. Therefore, ROS scavenging is a promising regenerative strategy. Intracellular ROS levels were assessed by DCFH-DA staining, and nuclei were labeled by DAPI staining (Figure 2E). The PA and SrPA groups showed significant ROS scavenging effects. Quantitative analysis revealed that while PA substantially decreased ROS levels, SrPA exhibited superior antioxidant activity, reducing the fluorescence intensity of ROS to levels comparable to those observed in the negative control group (Figure 2F).

### *In vitro* osteogenic differentiation performance

High ALP activity is a key early marker of osteogenic differentiation [39]. ALP staining was weaker in the control and PA groups on both days 7 and 14, indicating low ALP expression. In contrast, staining was intense in the Ca^2+^, Sr^2+^ and SrPA groups, which reflected substantially higher ALP levels and suggested strong early osteogenic potential (Figure 3A). Quantitative analysis further supported these observations (Figure 3C and D). ALP activity in all the experimental groups increased significantly on day 7, with the most pronounced effects observed in the Ca^2+^, Sr^2+^ and SrPA groups, with values of 2.13, 1.79 and 1.53 King units/gprot, respectively. By day 14, ALP activity further increased to 2.37, 2.03 and 1.95 King units/gprot. In the PA group, osteogenic activity was modest, with ALP levels increasing by approximately 0.24 King units/gprot relative to the control group on day 14. These quantitative results closely aligned with the ALP staining observations, indicating that Ca^2+^, Sr^2+^, and PA in the nnGS hydrogel effectively promoted early osteogenic differentiation of BMSCs.

**Figure 3:**
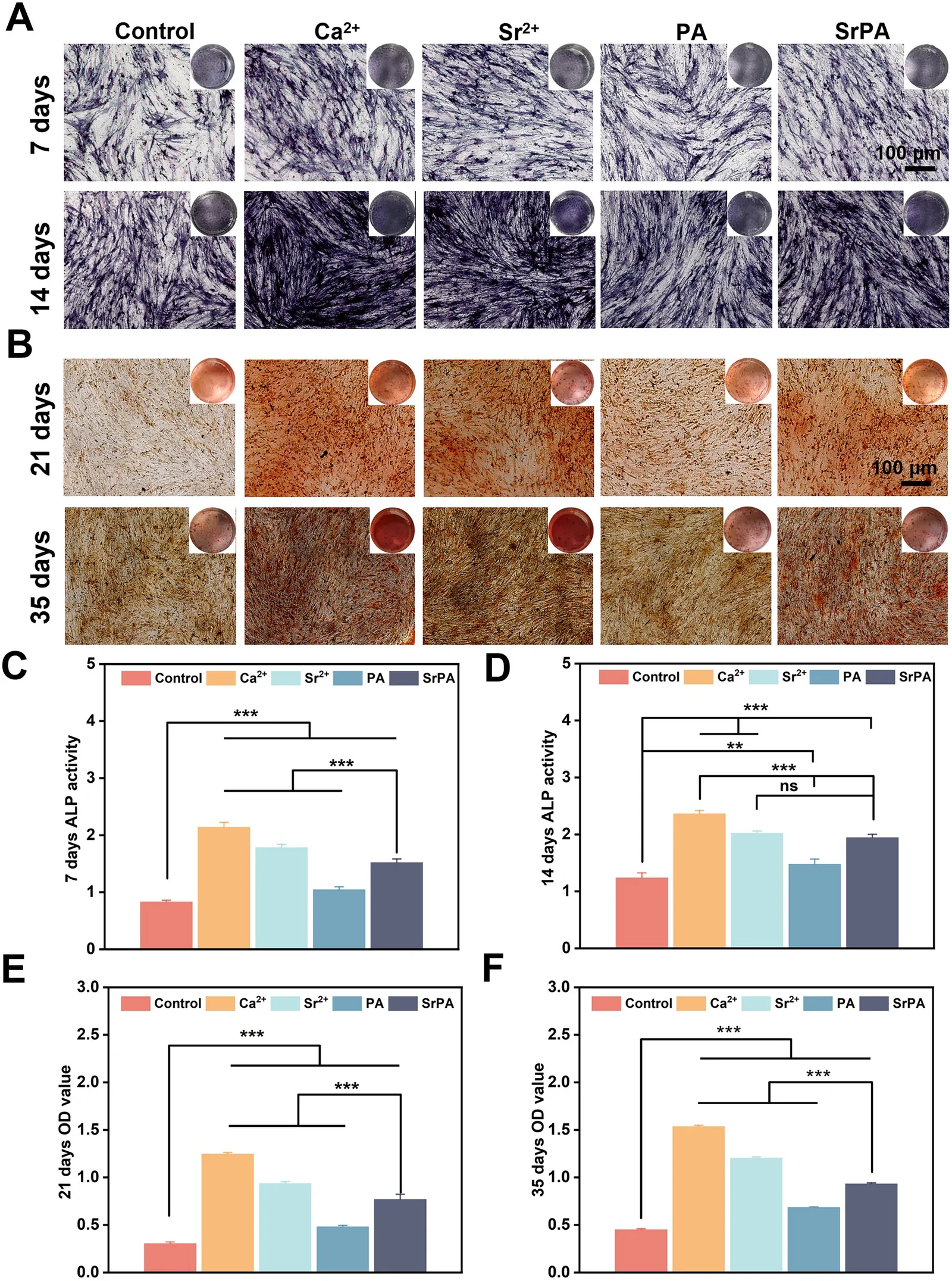
Osteogenic differentiation of BMSCs in vitro. (**A**) ALP staining after 7 and 14 days of culture; (**B**) ARS staining of mineralized nodules on days 21 and 35; quantitative analysis of (**C** and **D**) ALP activity and (**E** and **F**) Ca^2+^ nodule deposition at the indicated time points. (ns: not significant; *P < 0.05, **P < 0.01 and ***P < 0.001; n = 3; *P*-values were calculated using one-way ANOVA with Tukey’s multiple comparisons test).

To evaluate later stages of osteogenic maturation, ARS staining was performed to assess the formation of Ca^2+^ nodules, a hallmark of matrix mineralization [40]. On day 21, light red staining in the control and PA groups indicated minimal Ca^2+^ deposition (Figure 3B). In contrast, staining was intense in the Sr^2+^ and Ca^2+^ groups, reflecting abundant mineralization [30]. The staining intensity was slightly lower in the SrPA group than in the Sr^2+^ group, probably due to the gradual release of active components from the particles over time. By day 35, Ca^2+^ deposition had increased considerably across all the groups, as indicated by deeper red staining. Quantitative analysis confirmed these changes (Figure 3E and F). At both times, the formation of Ca^2+^ nodules significantly increased in all the experimental groups. The relative mineralization values for the Sr^2+^, Ca^2+^ and SrPA groups were 1.25, 0.94 and 0.77 at day 21, which increased to 1.54, 1.21 and 0.94 at day 35. A slight but detectable increase in mineralization was also observed in the PA group by day 35. These results collectively indicate that Sr^2+^, Ca^2+^ and PA released from the nnGS hydrogel effectively promote both intermediate and late osteogenic differentiation, thereby enhancing extracellular matrix mineralization.

### Sequencing analysis

To determine how Sr^2+^, PA and Ca^2+^ released from nnGS hydrogels drive the osteogenic differentiation of BMSCs, RNA-seq was performed. Principal component analysis (PCA) showed good clustering of biological replicates and revealed that Ca^2+^ and Sr^2+^ exerted relatively similar effects on the transcriptome profile (Figure 4A). Differentially expressed genes (DEGs) were identified based on thresholds of |log_2_FC| ≥ 1 and adjusted *P*-values ≤ 0.05. Volcano plots revealed that 1836 genes were upregulated and 2223 genes were downregulated in the Ca^2+^ group; 1437 genes were upregulated and 2103 genes were downregulated in the Sr^2+^ group; and 4795 genes were upregulated and 4396 genes were downregulated in the PA group (Figure 4B–D). Venn diagrams and heatmaps highlighted key osteogenic differentiation genes (screened based on NCBI Gene) that were commonly or uniquely regulated in the Ca^2+^, Sr^2+^ and PA groups compared with the control group. Several genes, including BMP-2, BMP-4, BMP-6, IBSP, FGF2, WNT9A and TGF-β_3_, were consistently influenced by all three treatments (Figure 4E–H). These genes have been reported to participate in the initiation and progression of osteogenic differentiation and maintenance of bone metabolic homeostasis [41, 42].

**Figure 4:**
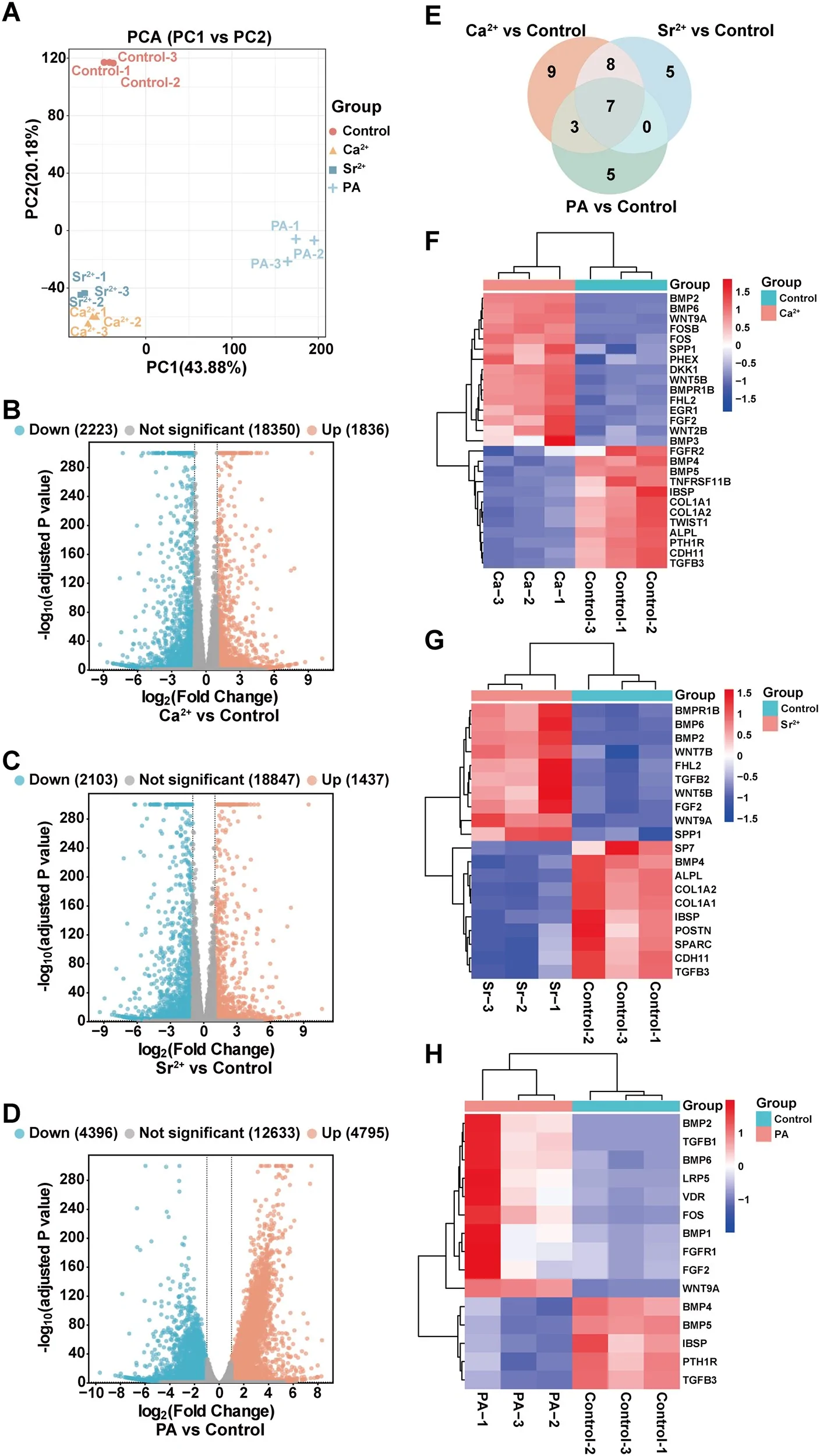
Transcriptome sequencing analysis of BMSCs treated with Ca^2+^, Sr^2+^ or PA. (**A**) PCA of the transcriptomes; (**B–D**) Volcano plots showing DEGs in the Ca^2+^, Sr^2+^ and PA groups compared with the control; (**E**) Venn diagram illustrating the overlap of osteoblast differentiation-related DEGs among the treatment groups. Heatmaps of osteoblast differentiation-related DEGs in the Ca^2+^ (**F**), Sr^2+^ (**G**) and PA (**H**) groups (*n* = 3).

Gene ontology enrichment analysis revealed that the Ca^2+^ group was significantly enriched in biological processes including skeletal system development, bone mineralization and osteogenic differentiation. Sr^2+^-treated cells were enriched in terms related to bone development, osteogenic differentiation and positive regulation of bone mineralization. Similarly, the capacity for osteogenic differentiation was enhanced in the PA group (Supplementary Figure S4A–C). Kyoto Encyclopedia of Genes and Genomes pathway analysis further revealed that DEGs in all three groups were notably enriched in pathways associated with osteogenic regulation. In the Ca^2+^ and Sr^2+^ groups, the peroxisome proliferator-activated receptor (PPAR) signaling pathway was among the most significantly enriched pathways, suggesting that it may represent a candidate pathway involved in ion-associated osteogenic regulation. Other pathways previously associated with osteogenic regulation, including Ca^2+^ signaling, Wnt, PI3K-Akt and JAK-STAT signaling, were also enriched, providing potential directions for future mechanistic validation (Supplementary Figure S4D and E) [43]. In the PA-treated group, the immune regulation related TNF signaling pathway was the most enriched pathway, suggesting the possible involvement of TNF-associated signaling in the cellular response to PA (Supplementary Figure S4F).

### qRT-PCR analysis

The osteogenic effects of Ca^2+^, PA and Sr^2+^ were further evaluated by qRT-PCR (Figure 5A–D). PA significantly upregulated the expression of COL-1, indicating enhanced collagen synthesis, but did not promote the expression of OCN, RUNX2 or BMP-2. In contrast, Ca^2+^ significantly enhanced the expression of RUNX2, BMP-2 and OCN, indicating a pronounced osteogenic effect. Sr^2+^ significantly increased OCN expression, whereas its effects on RUNX2 and BMP-2 did not reach statistical significance. Collectively, these findings suggest that Ca^2+^ and Sr^2+^ exert distinct but complementary effects on osteogenic differentiation, potentially compensating for the relatively limited osteogenic activity of PA [44].

**Figure 5:**
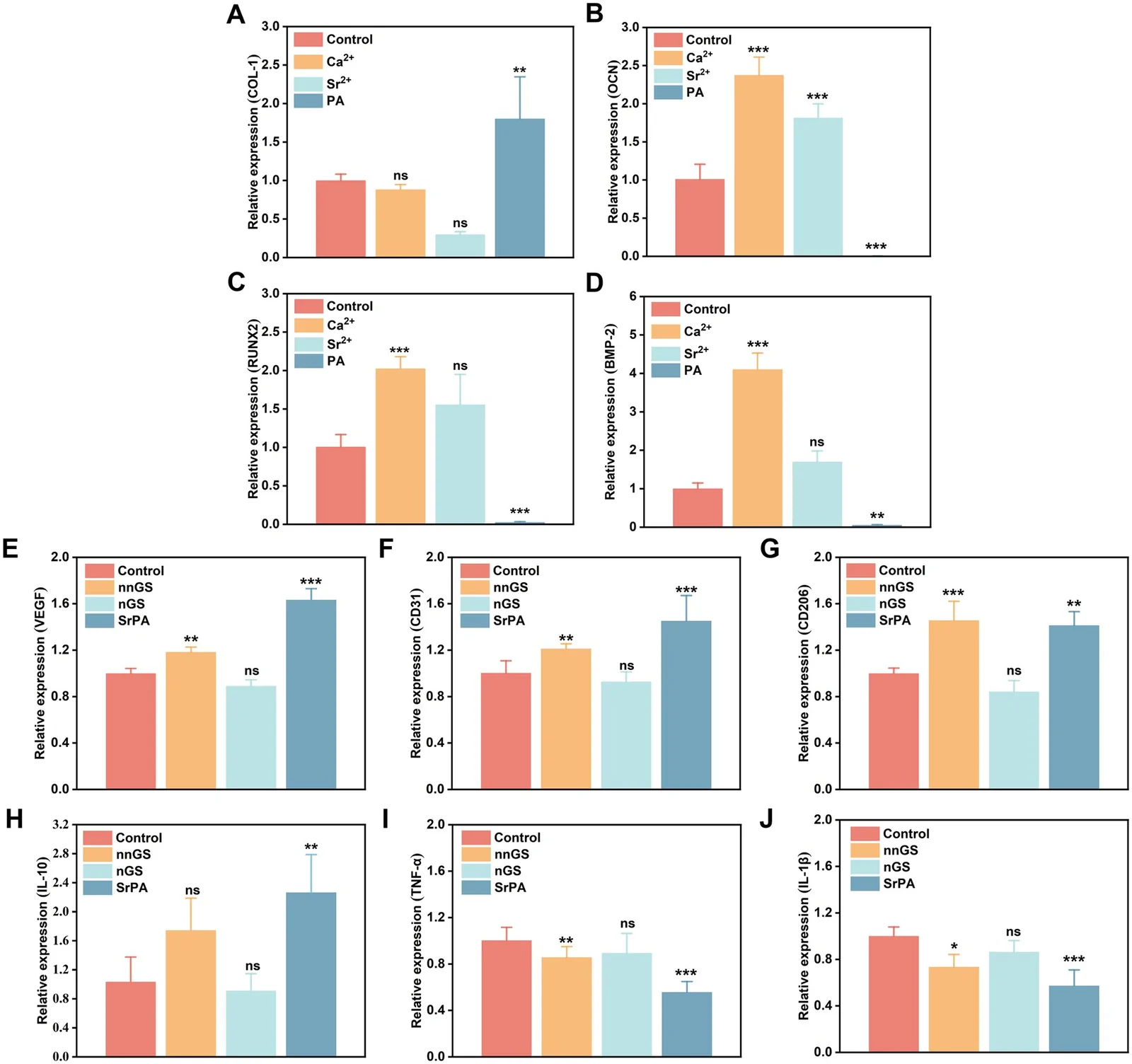
Relative mRNA expression of osteogenic genes in BMSCs treated with Ca^2+^, Sr^2+^ and PA (**A–D**). Relative mRNA expression of genes associated with angiogenesis in HUVECs (**E** and **F**) and inflammation in RAW 264.7 macrophages (**G–J**) treated with nnGS, nGS and SrPA (ns: not significant; *P < 0.05, **P < 0.01 and ***P < 0.001 vs. the control group; *n* = 3; *P*-values were calculated using one-way ANOVA with Dunnett’s multiple comparisons test).

By loading SrPA particles onto the nGS hydrogel, the nnGS hydrogel was obtained. qRT-PCR analysis of HUVECs revealed that compared with the control, nnGS and free SrPA significantly upregulated the expression of VEGF and CD31, with SrPA displaying the strongest pro-angiogenic activity (Figure 5E and F). In the nnGS hydrogels, sustained Sr^2+^ release following SrPA decomposition helped maintain high expression levels of these factors. Based on previous reports, sustained Sr^2+^ release may contribute to the upregulation of angiogenesis-related genes in HUVECs, potentially involving MAPK/ERK and PI3K/Akt-related signaling pathways [45].

Analysis of inflammatory gene expression in RAW264.7 macrophages revealed varied immunomodulatory effects among the groups (Figure 5G–J). While expression of pro-inflammatory (TNF-α and IL-1β) and anti-inflammatory (CD206 and IL-10) markers did not significantly differ between the nGS and control groups, both nnGS and SrPA significantly increased anti-inflammatory markers and inhibited pro-inflammatory markers. These findings suggest that PA release from SrPA decomposition exerts potent anti-inflammatory effects.

### Characterization of the hydrogels

Primary cross-linking of the hydrogel started in the acidic environment provided by Glu, where HAp dissolved to release Ca^2+^, which facilitated the cross-linking of alginate chains. Mechanical compression tests revealed that the primarily cross-linked hydrogel had a low compressive strength of approximately 4 kPa at 50% strain, indicating that the network was loosely interconnected (Figure 6A). Secondary cross-linking in CaCl_2_ solution substantially increased the mechanical strength. Compared with primary cross-linking, the hydrogel cavities after secondary cross-linking became denser (Supplementary Figure S5). At 60% strain, nnGS-12.5 achieved the highest compressive strength (170.8 kPa), which was significantly better than the nGS group (139.9 kPa) and the nnGS-25 group (145.2 kPa). SEM images revealed uniform particle distribution on the nnGS-12.5 surface, whereas significant agglomeration was observed on the nnGS-25 hydrogel (Supplementary Figure S6). The lower strength of nnGS-25 may be due to particle agglomeration decreasing surface area and interfacial interactions, as well as competitive coordination between the Ca^2+^ and PA catechol groups disrupting alginate cross-linking. Although nnGS hydrogels cannot fully replicate the mechanical function of mature trabecular bone, they closely resemble osteogenic granulation tissue formed during early bone defect repair. With subsequent new bone deposition and enhanced mineralization, the mechanical properties of the overall tissue improve, gradually achieving functional restoration of the defect.

**Figure 6:**
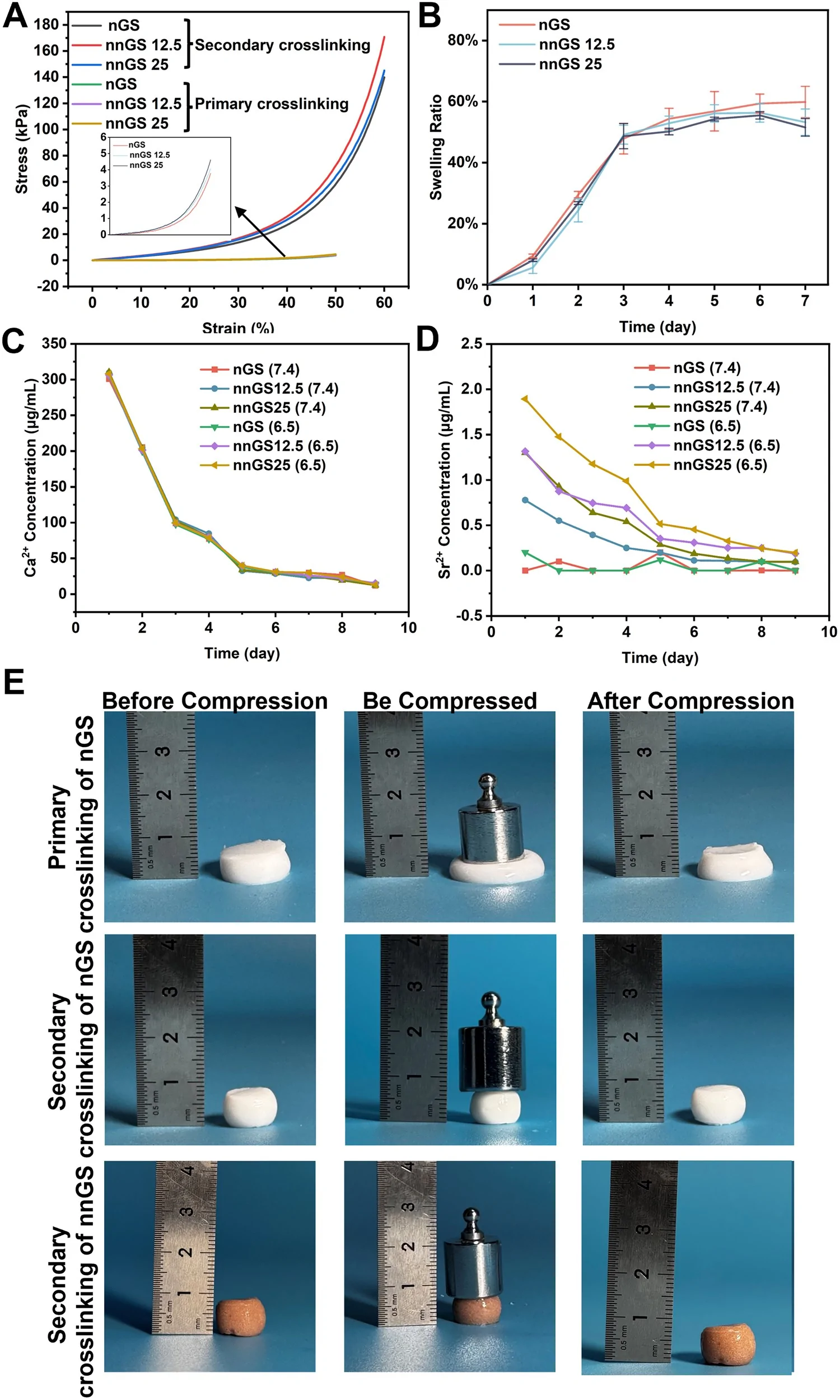
Mechanical and physicochemical characterization of hydrogels. (**A**) Hydrogel compressive strength test; (**B**) Swelling kinetics of hydrogels with different compositions (*n* = 3); Interval release profiles of (**C**) Ca^2+^ and (**D**) Sr^2+^ from the hydrogels over time; (**E**) Representative images illustrating the deformation and recovery of hydrogels under static loading.

Swelling behavior analysis indicated that secondary cross-linking promoted the formation of a dense hydrogel layer, with the alginate surface reaching swelling equilibrium within 3 days (Figure 6B). Variations in pH and SrPA incorporation did not significantly alter the Ca^2+^ release kinetics; Ca^2+^ were released rapidly within the first three days. In contrast, the release of Sr^2+^ and PA depended on both pH and SrPA concentration, demonstrating sustained release over 7 days, with quantities increasing progressively at higher SrPA concentrations, particularly under acidic conditions (Figure 6C and D, and Supplementary Figure S7). This differential release behavior is governed by distinct mechanisms: Ca^2+^ release is driven primarily by hydrogel swelling, whereas Sr^2+^ and PA release results from the gradual dissociation of SrPA under weakly acidic conditions.

Compression recovery tests illustrated the impact of secondary cross-linking (Figure 6E). The singly cross-linked nGS hydrogel showed poor recovery (71% ± 1.2%), whereas the doubly cross-linked nGS and nnGS hydrogels exhibited significantly improved resistance to deformation and recovery rates (87% ± 2.7% and 92% ± 3.5%, respectively). EDS analysis confirmed SrPA incorporation, with 19.55% ± 3.8% Sr^2+^ detected in the nnGS hydrogel (Supplementary Table S1). Live/dead staining revealed that the cell morphology did not change significantly after co-culture with primary and secondary cross-linked hydrogels. CCK-8 results showed cell viability similar to the control group, indicating good cytocompatibility of the nGS hydrogel (Supplementary Figure S8).

### Micro-CT and histological analysis

Micro-CT and histological analysis of rat calvarial defect sections at 6 and 12 weeks post-implantation revealed enhanced bone regeneration in the nnGS group (Figure 7A). Quantitative micro-CT revealed that nGS exceeded the HAp and control treatments in terms of BMD and BV/TV, whereas nnGS achieved the highest values at both time points (Figure 7B and C).

**Figure 7:**
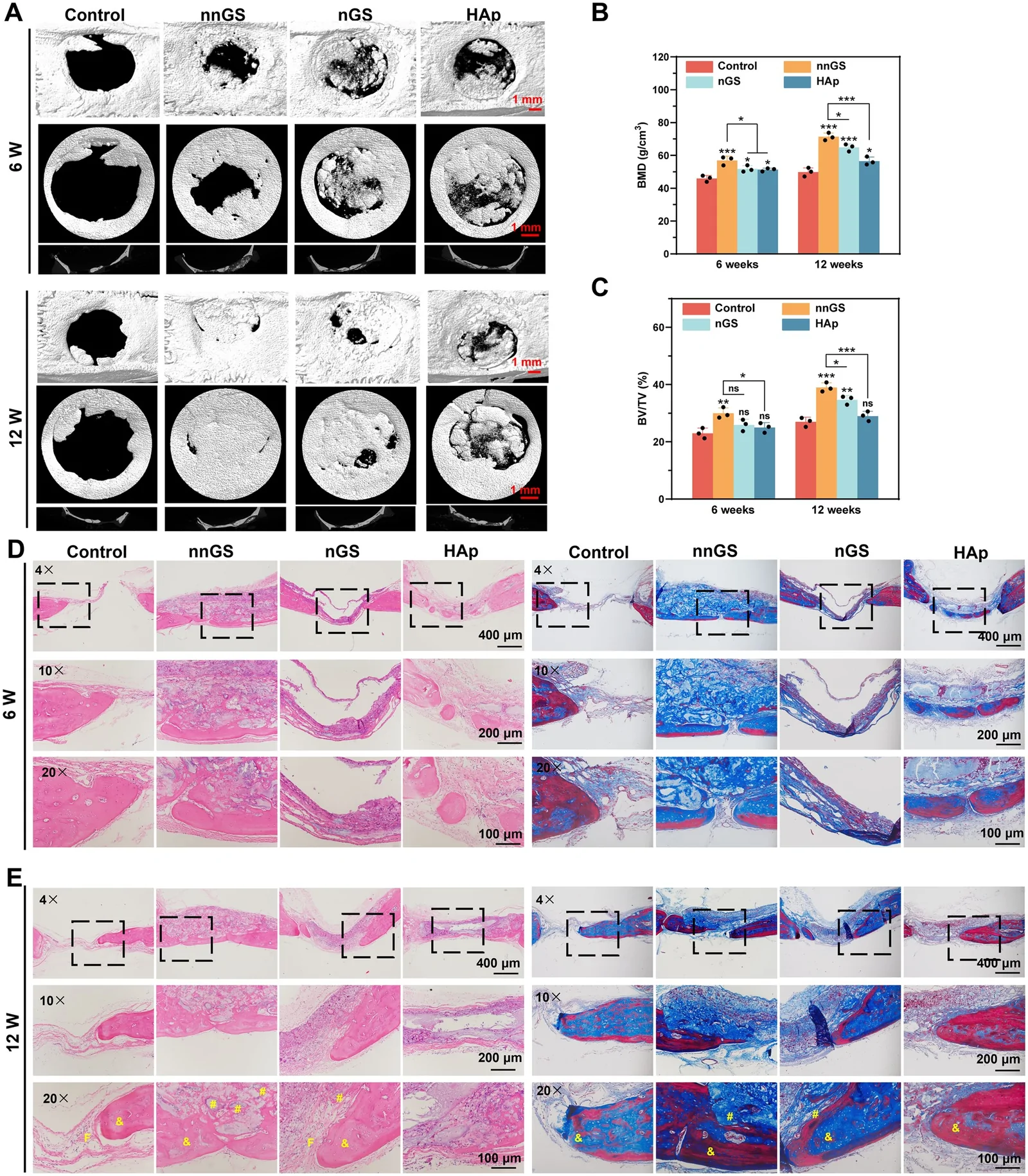
Evaluation of *in vivo* bone regeneration in a rat calvarial defect model. (**A**) Representative micro-CT reconstructions at weeks 6 and 12 post-surgery; (**B** and **C**) Quantitative analysis of BMD and BV/TV at each time point (ns: not significant; *P < 0.05, **P < 0.01 and ***P < 0.001; n = 3; P-values were calculated using one-way ANOVA with Tukey’s multiple comparisons test); (**D** and **E**) H&E and Masson’s trichrome staining at weeks 6 and 12 (ampersand indicates native bone, F indicates fibrous tissue and hash indicates new bone).

The regeneration process was visualized by H&E staining (Figure 7D and E). At week 6, the nnGS group exhibited dense fibrous tissue with reduced fibrous spacing and early evidence of bone formation. By week 12, continuous trabecular bone bridged the defect and integrated seamlessly with the host margins. This accelerated bone regeneration was associated with the combined effects of the released Sr^2+^ and PA, which collectively enhanced angiogenesis, suppressed inflammation and stimulated the proliferation of osteoblasts. Masson’s trichrome staining provided additional insight into matrix maturation. At 6 weeks, the fibrous tissue spacing in the nnGS group decreased, resulting in the formation of a dense collagen matrix. Clear blue staining revealed that the newly formed collagen filled the defect area and fused well with the host bone. Compared to the nnGS group, the nGS group showed less collagen deposition and larger gaps between collagen fibers. At 12 weeks, the nnGS group exhibited thick, well-aligned collagen bundles and nascent osteons, corroborating active mineralization.

### Immunofluorescence staining analysis

To evaluate the osteogenic, immunomodulatory and angiogenic responses of the implanted hydrogel *in vivo*, immunofluorescence staining of bone defect sections was performed (Figure 8A). RUNX2, a key early transcription factor influencing osteoblast differentiation, exhibited the strongest fluorescence signal in the nnGS group at weeks 6 and 12, while signals in the nGS and HAp groups were substantially weaker, and the control group showed the lowest intensity. Quantitative analysis confirmed that RUNX2 expression was significantly greater in the nnGS group than in all other groups at both time points (Figure 8B). ALP expression was consistent with RUNX2. At week 6, extensive and intense ALP-positive signals were observed in the nnGS group, with a similar trend at week 12. However, the expression in the nGS group was somewhat weaker than that in the nnGS group. Compared with those in the other groups, ALP levels in the nnGS group were significantly greater at both time points (Figure 8C). OCN, a crucial marker of late-stage osteogenesis and mineralization, better reflects newly formed bone maturity. At week 12, the most concentrated and continuous OCN signals were detected in the nnGS group. Quantitative analysis revealed that the expression of OCN was significantly greater in the nnGS group than in other groups at weeks 6 and 12; the difference was more pronounced at week 12 (Figure 8D). These findings indicate that nnGS increased osteogenic-marker expression and suggest matrix maturation.

**Figure 8:**
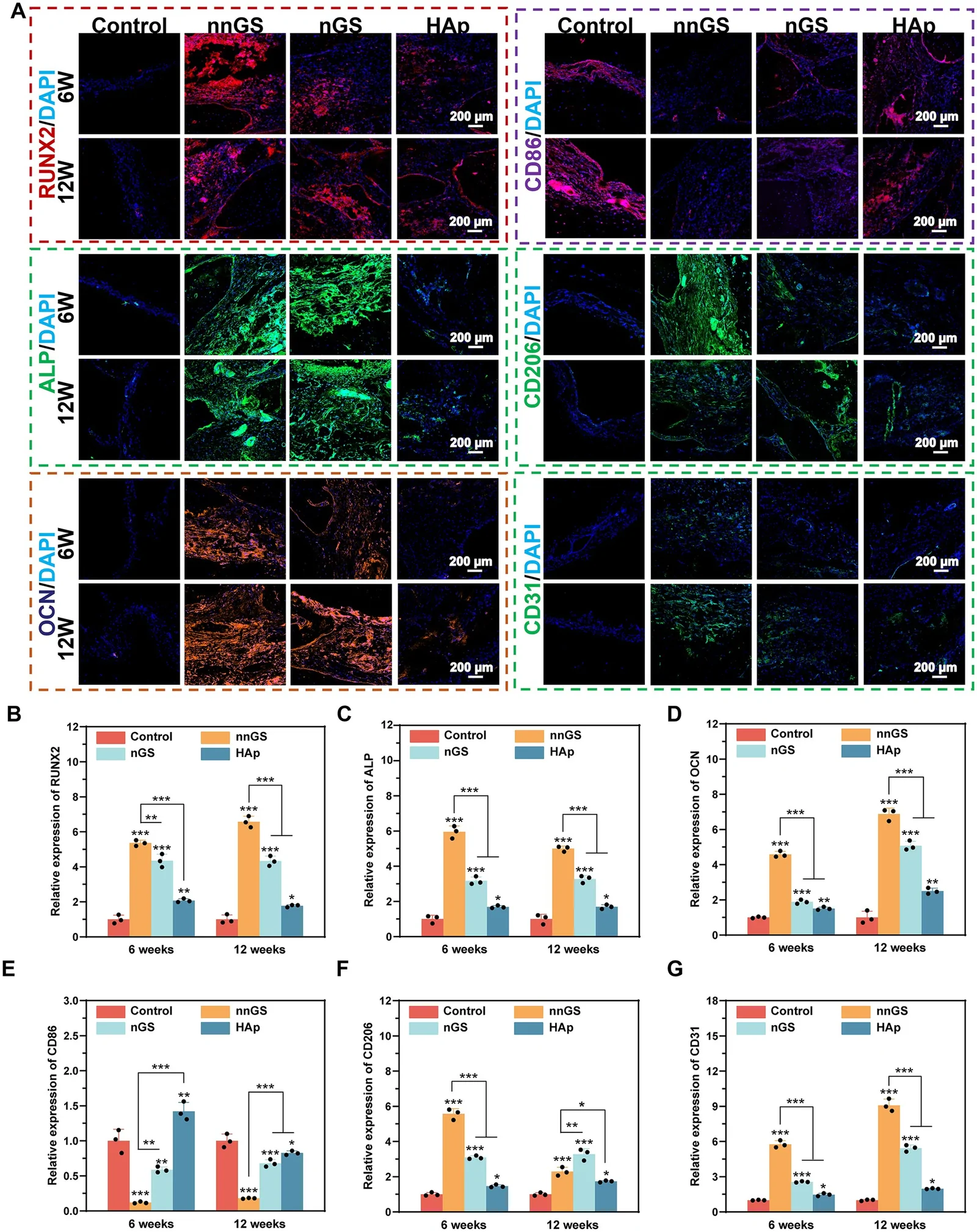
*In vivo* immunofluorescence analysis of bone regeneration, immune response and angiogenesis at the defect site. (**A**) Immunofluorescence staining images of osteogenic markers (RUNX2, ALP and OCN), pro-inflammatory (CD86) and repair-associated (CD206) markers, and the endothelial marker CD31; (**B–G**) quantitative analysis of the fluorescence intensity of each marker (*P < 0.05, **P < 0.01 and ***P < 0.001; *n* = 3; *P*-values were calculated using one-way ANOVA with Tukey’s multiple comparisons test).

CD86 and CD206 expression revealed the regulatory effect of different materials on the inflammatory microenvironment. Immunofluorescence analysis of CD86 demonstrated that the HAp group exhibited relatively higher CD86 expression at both weeks 6 and 12, whereas the nnGS group had significantly lower CD86 expression. Quantitative analysis revealed that CD86 expression significantly decreased in the nnGS group, indicating that nnGS alleviated the pro-inflammatory response (Figure 8E). In contrast, the expression of CD206 showed the opposite pattern. Immunofluorescence and quantitative analyses revealed that the expression of CD206 was the highest in the nnGS group at week 6, significantly greater than in the nGS, HAp, and control groups. These findings indicate that nnGS was associated with altered expression of pro-inflammatory and repair-associated markers within the defect microenvironment (Figure 8F). CD31, a critical marker of endothelial cells and neovascularization, reflects the ability of a material to regulate vascularization. Immunofluorescence analysis revealed that distinct CD31-positive signals were already present in the nnGS group by week 6, and by week 12, denser and more continuous vascular-like structures had formed. A promoting effect was also found in the nGS group, but to a lesser extent. Quantitative analysis revealed that the expression of CD31 was significantly greater in the nnGS group than in all other groups at both time points, suggesting that nnGS supported angiogenesis responses during bone defect repair (Figure 8G). In summary, these immunofluorescence assays suggest that the nnGS hydrogel has favorable pro-angiogenic and immunomodulatory effects, providing the nutritional and microenvironmental support required for bone defect repair.

Histological staining of the heart, liver, spleen, lungs and kidneys revealed that in both the nGS and nnGS groups, myocardial fibers were well organized with no inflammatory cell infiltration; hepatocytes showed no signs of injury; the spleen exhibited no structural damage; alveolar structures were intact and renal tubular epithelial cell integrity was preserved (Supplementary Figure S9). Therefore, no significant histopathological abnormalities were observed under the testing conditions.

## Discussion

In this study, we developed a SrPA-loaded mineralized alginate/HAp hydrogel (nnGS) to regulate the osteogenic microenvironment through coordinated control of oxidative stress, inflammation, vascularization and bone formation. The dual-cross-linked hydrogel provided a stable 3D matrix, while the incorporation of SrPA introduced an additional bioactive component that could be retained within the hydrogel and gradually dissociate into Sr^2+^ and PA. This design allowed the structural function of the hydrogel and the biological functions of the coordination particles to be integrated within one material system. Compared with previously reported alginate/HAp composite hydrogel and metal–polyphenol coordination systems, the nnGS hydrogel offers a distinctive advantage by combining a mechanically reinforced mineralized matrix with sustained co-delivery of Sr^2+^, PA and Ca^2+^, enabling simultaneous modulation of inflammation, angiogenesis and osteogenesis within a single hydrogel [46–49].

The addition of SrPA enhanced the biological activity of the hydrogel in several complementary aspects. PA was associated with reduced oxidative stress-associated fluorescence and modulation of inflammatory-marker expression, suggesting that the coordination particles may help create a more favorable immune microenvironment. Meanwhile, the released Sr^2+^ supported endothelial cell migration and tube formation, indicating a potential contribution to vascularization. Sr^2+^, PA and Ca^2+^ also influenced osteogenic-marker expression and extracellular matrix mineralization in BMSCs. These findings suggest that SrPA does not function as a single-purpose additive; rather, its incorporation provides a coordinated reservoir of metal ions and polyphenols that may regulate multiple biological processes involved in bone repair. Notably, the dose of SrPA used in this study was selected based on preliminary cytocompatibility screening and falls within the ranges previously reported as safe for Sr^2+^ and PA. The cumulative released concentrations of Ca^2+^ and Sr^2+^ from the nnGS hydrogel remained well below reported cytotoxic thresholds for BMSCs and other cell types [50, 51], and no obvious histopathological abnormalities were observed in major organs.

The *in vivo* findings further support the regenerative potential of nnGS. Compared with the control group, nnGS was associated with improved new bone formation, increased expression of osteogenic and vascularization-related markers, and a more favorable inflammatory-marker profile. The enhanced bone repair may result from the combined effects of the mineralized hydrogel matrix, sustained release of Sr^2+^ and PA, and the improved cellular microenvironment created by the hydrogel. However, the transcriptomic results should be regarded as exploratory evidence for candidate osteogenic and inflammation-related pathways rather than definitive proof of pathway activation.

Despite these promising findings, this study has several limitations. First, hydrogel-level single-component control groups, such as nGS + Sr^2+^ and nGS + PA, were not included. Therefore, the individual contributions of Sr^2+^ release, PA release, SrPA coordination and changes in hydrogel microstructure or mechanical properties cannot be clearly distinguished. Although the free-component experiments support the biological plausibility of these contributions, they do not substitute for hydrogel-level component-isolation controls. Second, the current calvarial defect model is non-load bearing and was performed in rats. Future studies should evaluate nnGS in clinically relevant load-bearing bone defect models, preferably in larger animals and further assess its mechanical stability, degradation behavior and the match between material degradation and new bone formation rates, as well as host immune responses over extended periods. Third, the *in vivo* experiments included only three independent animals per group at each time point. Although consistent regenerative trends were observed across the evaluated parameters, the limited sample size may reduce the precision of variance and effect-size estimates. Accordingly, these *in vivo* findings should be considered preliminary and interpreted with appropriate caution, and they require further validation in larger, adequately powered animal studies.

## Conclusions

In this study, we constructed a biomimetic composite hydrogel (nnGS) that promotes bone repair through the coordinated effects of inflammation, angiogenesis and osteogenesis. A key innovation was the development of SrPA particles, which dissociate in the bone defect microenvironment to release PA and Sr^2+^, effectively mitigating inflammation, scavenging ROS and supporting angiogenic responses. Along with Ca^2+^ from the hydrogel matrix, these components exert combined effects on bone regeneration. *In vivo* experiments demonstrated that the nnGS hydrogel significantly promoted bone repair, as indicated by the substantially upregulated expression of osteogenic markers, reduced inflammatory response and upregulated expression of vascularization markers.

## Supplementary Material

rbag185_Supplementary_Data

## Data Availability

The data that support the findings of this study are available from the corresponding author on reasonable request.
