# Supplementary material for: Biomimetic mineralized hydrogels loaded with strontium-proanthocyanidins coordination particles for bone defect repair
Source: Regen Biomater. 2026 Sep 7;13:rbag185. doi: 10.1093/rb/rbag185 (PMC13633663; doi:10.1093/rb/rbag185)
Supplement: Supplementary file 1 [file rbag185_supplementary_data.docx]

**Biomimetic mineralized hydrogels loaded with strontium-proanthocyanidins coordination particles for bone defect repair**

Fengkai Yang^1#^, Yijian Xie^3^^#^, Chengye Jiang^3^, Xiang Jia^3^, Xinshuo Liu^3^, Lihua Li^3^*, Yifan Zhang^2,3^*, Mingyan Zhao^1^*, Changren Zhou^3^

1. Stem Cell Research and Cellular Therapy Center, Affiliated Hospital of Guangdong Medical University, Zhanjiang 524001, China.
2. Institute of Dermatology and Venereal Diseases, Affiliated Hospital of Guangdong Medical University, Zhanjiang, 524001, China.
3. Department of Materials Science and Engineering, Engineering Research Center of Artificial Organs and Materials, Jinan University, Guangzhou 510632, China.

\# These authors contributed equally to this work.

*Corresponding authors:

Yifan Zhang, Email address: yifanzhang0916@163.com

Mingyan Zhao, Email address: mingyan.zhao@gdmu.edu.cn

Lihua Li, Email address: tlihuali@jnu.edu.cn

## **2.3 Preparation and characterization of hydrogels**

**2.3.1 Preparation of hydrogel****s**

Glutamate (Glu, 200 mg) was first dissolved in 100 mL of deionized water. After complete dissolution, sodium alginate (2 g) was slowly added and stirred for 12 h to obtain a homogeneous Glu/SA mixture. Separately, HAp (0.6 g) was dispersed in 5 mL of deionized water by ultrasonication and subsequently mixed with 15 mL of the Glu/SA solution under vigorous mechanical stirring for 1 min[1, 2]. The resulting mixture was cast into a mold and incubated at 4 ℃ for 12 h to form the primarily cross-linked nGS hydrogel. After demolding, the hydrogel was immersed in a 4% (w/v) calcium chloride solution with stirring for 2 h for secondary cross-linking, yielding the final nGS hydrogel.

For SrPA-doped hydrogels, SrPA particles (250 μg or 500 μg) were premixed with HAp (0.6 g) and ultrasonically dispersed in 5 mL of deionized water. The HAp-SrPA dispersion was then blended with the Glu/SA solution as described above, giving final SrPA concentrations of 12.5 μg/mL and 25 μg/mL, respectively. These hydrogels were designated as nnGS-12.5 and nnGS-25.

### **2.3.2 Characterization of the hydrogels**

The compressive performances of the primary and secondary cross-linked nGS and nnGS hydrogels (cylindrical specimens: φ 10 mm × 10 mm) were evaluated using a universal tensile testing machine (CMT6103, MTS, USA). A compressive load was applied at a constant rate of 3 mm/min until the deformation reached 60%. To visually observe deformation behavior, a 20 g weight was placed on the hydrogels in a separate test. Images were taken before, during, and after compression to compare the mechanical resilience of hydrogels under different cross-linking conditions.

The swelling behavior of the secondary cross-linked nnGS hydrogel was investigated by incubating individual samples in a 12-well plate with 3 mL of sterile PBS per well (n=3). At predetermined time intervals, the hydrogels were removed, and surface moisture was blotted with filter paper before weighing (recorded as Wt, where W_0_ is the initial weight). The swelling ratio (SR) was calculated as the equation (2):

SR (%) = (W_t_-W_0_)/W_0_ ×100%	(2)

The morphology of nnGS-12.5 and nnGS-25 hydrogels, as well as the nnGS-12.5 hydrogel before and after two cross-linking steps, was detected using scanning electron microscopy (SEM, TESCAN MIRA LMS, Czechia).

To evaluate the release profiles of Ca^2+^ and Sr^2+^, nnGS hydrogel samples were immersed in 50 mL centrifuge tubes containing 10 mL of sterile PBS (pH=7.4 and 6.5, n=3), the release medium was collected every 24 h. After each collection, 10 mL of fresh PBS was replenished. The concentrations of released Ca^2+^ and Sr^2+^ were quantified by inductively coupled plasma optical emission spectrometry (ICP, PerkinElmer 8300, USA).

The release of PA from the nnGS hydrogel was measured using the same collection method. The PA concentration was determined by ultraviolet spectrophotometry at 278 nm. Blank nGS hydrogel samples without PA were incubated using the same collection procedure, and their absorbance at 278 nm was measured at each corresponding time point. The absorbance contributed by other hydrogel components was subtracted from the absorbance of the PA containing samples. A standard curve was established between PA concentration and absorbance, yielding the equation y = 0.0108x + 0.0025 (R² = 0.9999). The amount of PA released at each time point was calculated from this standard curve, and the release profile was plotted accordingly.

The cytocompatibility of nGS hydrogel was tested using a CCK-8 assay and live/dead staining. BMSCs were co-cultured with primary and secondary cross-linked nGS hydrogel for 1, 3, and 5 days. Cell viability was measured at each time point using CCK-8 assay, and live/dead staining was observed under a fluorescence microscopy.

### **2.3.3 *In vitro* osteogenic characterization**

The experiment included five groups: Control, Ca^2+^, Sr^2+^, PA, and SrPA. BMSCs were seeded in 12-well plates at a density of 5×10^4^ cells per well. After 3 days of culture, the cell culture medium was replaced with osteogenic induction medium (Oricell, HUXMX-90021) specific to each group. The Ca^2+^ (300 μg/mL CaCl_2_), Sr^2+^ (5.3 μg/mL SrCl_2_), and PA (10 μg/mL) groups were treated at their respective maximum safe concentrations, while the SrPA group received a concentration of 12.5 μg/mL. The osteogenic medium was refreshed every 2 days[3, 4].

After 7 and 14 days of treatment, ALP staining was performed following the instructions of the BCIP/NBT chromogenic kit, and the cells were observed and photographed under a microscope (Olympus BX53, Japan). In parallel, cells under the same conditions in separate wells were lysed with 500 μL of 1% Triton X-100 (Sigma-Aldrich, USA) at room temperature for 30 min. ALP activity in the lysates was then quantified using an ALP assay kit and normalized to total protein content determined by a BCA protein assay kit.

BMSCs were seeded in 12-well plates at a density of 5×10⁴ cells per well. After 3 days of culture, the cell culture medium was switched to osteogenic induction media corresponding to each group (Control, Ca^2+^, Sr^2+^, PA, and SrPA, at the concentrations indicated above), and the media were refreshed every 2 days. Following 21 and 35 days of induction, the cells were fixed and stained with Alizarin Red S (ARS) to visualize calcium nodules formation. For quantification, the stained nodule was dissolved in 500 μL of 10% (w/v) cetylpyridinium chloride (Macklin) solution and incubated at room temperature for 30 min. The absorbance of the solution was then measured at 562 nm using a microplate reader (Thermo Fisher Scientific).

### **2.3.4 Quantitative real-time PCR (qRT-PCR) analysis**

To investigate osteogenic differentiation, BMSCs were seeded in 6-well plates at 5×10⁴ cells per well. After culture for 24 h, the cells were treated with Ca^2+^, Sr^2+^, or PA at the same concentrations as described above (Section 2.3.3) for 7 days before harvest.

To evaluate the pro-angiogenic effects, HUVECs were seeded in 6-well plates at 5×10⁴ cells per well. After 24 h of adhesion, the culture medium was replaced with fresh medium containing nnGS, nGS, or SrPA (12.5 μg/mL) in transwell inserts (6-well plates, aperture: 8 μm, diameter: 24 mm, Beijing LABSELECT), and the cells were cultured for an additional 7 days before harvest for gene expression analysis.

To assess anti-inflammatory effects, RAW264.7 macrophages (Procell, CL-0190) were seeded in 6-well plates at a density of 5×10⁴ cells per well and cultured for 24 hours. The cells were stimulated with lipopolysaccharide (LPS, 1 μg/mL, Sigma-Aldrich) for 12 h to induce M1 polarization. After LPS stimulation, the culture medium was replaced with fresh medium, and the cells were co-cultured with nnGS, nGS, or SrPA (12.5 μg/mL) in transwell inserts for an additional 24 h before harvest for gene expression analysis[5].

Total RNA was extracted from all harvested cells using trizol reagent (Macklin). After reverse transcription, the cDNA samples were analyzed using a qRT-PCR instrument (Applied Biosystems) with SYBR Green dye. Relative gene expression was calculated using the 2^-ΔΔCt^ method and normalized to the respective Control groups. The primer sequences are shown in Tables S2, S3, and S4.

### **2.3.5 RNA sequencing**

BMSCs were seeded in 6-well plates at a density of 5×10^4^ cells per well and cultured for 3 days. The cells were then divided into four groups (Control, Ca^2+^, Sr^2+^ and PA) and cultured for another 14 days. Three independent biological replicates were included per group. After incubation, cells from each group were collected, labeled, flash-frozen in liquid nitrogen, and stored at -80 °C until further processing. Transcriptome sequencing and bioinformatic analysis were performed by Wuhan Kindstar Sequenon Biotechnology Co., Ltd. The RNA sequencing data generated in this study has been deposited in the NCBI Gene Expression Omnibus (GEO) under accession number GSE343848.

RNA purity, concentration, and integrity were assessed using a NanoDrop spectrophotometer (ThermoFisher Scientific), Qubit 4.0 fluorometer (Thermo Fisher Scientific, Q33238), and Qsep 400 (BiOptic, China), respectively. Only RNA samples meeting the predefined quality-control criteria, including an RNA Quality Number (RQN) ≥ 7.0, an A260/A280 ratio of 1.8-2.1, and a 28S/18S rRNA ratio ≥ 1.5, were used for library preparation. Libraries were prepared using the VAHTS Universal V8 RNA-seq Library Prep Kit for Illumina (Vazyme) and sequenced on the Illumina X Plus platform with 150-bp paired-end reads. Raw sequencing reads were quality-controlled using fastp (v0.23.4) by removing adapter-containing, poly-N-containing, and low-quality reads, with Q20, Q30, GC content, and sequence-duplication levels assessed. Clean reads were aligned to the human GRCh38 (hg38) reference genome using STAR (v2.7.8a). Gene-level counts were generated using featureCounts (v2.0.4). Raw gene-level counts were normalized using the default size-factor normalization implemented in DESeq2 (v1.46.0), and differential-expression analysis was subsequently performed based on the negative-binomial model. P values were adjusted using the Benjamini-Hochberg method, and genes with adjusted P value ≤ 0.05 and |log₂(FC)| ≥ 1.0 were defined as DEGs. Sample correlation analysis and principal component analysis (PCA) were performed to assess the overall similarity and clustering of biological replicates. GO enrichment analysis of the identified DEGs was performed using clusterProfiler, and KEGG pathway enrichment analysis was performed using KOBAS and clusterProfiler.

**Figure S1.** Schematic illustration of nnGS hydrogel preparation and its application in a rat calvarial defect model.

**Figure S2.** Cytocompatibility of BMSCs after treatment with different concentrations of SrPA for 24 h (ns: not significant; *P < 0.05 vs Control group; n=3; P-values were calculated using one-way ANOVA with Dunnett’s multiple comparisons test).

**Figure S3.** Representative images of *in vitro* tube formation by HUVECs incubated with Sr^2+^, PA, and SrPA for 6 h (**P < 0.01, ***P < 0.001 vs Control group; n=3; P-values were calculated using one-way ANOVA with Dunnett’s multiple comparisons test).

**Figure S4.** GO enrichment analysis of biological processes for DEGs in the Ca^2+^ (A), Sr^2+^ (B), and PA (C) groups; KEGG pathway enrichment analysis for the DEGs in the Ca^2+^ (D), Sr^2+^ (E), PA (F) groups compared with the Control (n=3).

**Figure S5.** SEM images of nnGS hydrogel for primary crosslinking and secondary crosslinking.

**Figure S6.** SEM images of nnGS-12.5 (A) and nnGS-25 (B) hydrogels.

**Figure S7.** Interval release profiles of PA from hydrogels over time.

**Figure S8.** Cytocompatibility of nGS hydrogel (ns: not significant; n=3; P-values were calculated using one-way ANOVA with Tukey’s multiple comparisons test).

**Figure S9.** H&E staining of heart, liver, spleen, lung, and kidney sections from rats at 12 weeks post-surgery.

**Table S1** Elemental quantification of nGS hydrogel and nnGS hydrogels by EDS

|  | C (%) | N (%) | O (%) | P (%) | Ca (%) | Sr (%) |
| --- | --- | --- | --- | --- | --- | --- |
| nGS | 11.62±1.3 | 0.81±0.3 | 30.19±2.7 | 21.10±1.9 | 36.28±3.7 | 0 |
| nnGS | 11.28±1.5 | 1.94±0.3 | 28.58±2.5 | 13.36±1.9 | 25.38±2.3 | 19.55±3.8 |

**Table S2** Primers Sequences for osteogenic differentiation-related genes

| Primer | Forward Sequence（5’-3’） | Reverse Sequence（5’-3’） |
| --- | --- | --- |
| COL-1 | CAGCCGCTTCACCTACAGC | TTTTGTATTCAATCACTGTCTTGCC |
| OCN | CTCACACTCCTCGCCCTATT | CTTACTGCGGCTGCTGTGAC |
| RUNX2 | CCGCACGACAACCGCACCAT | CGCTCCGGCCCACAAATCTC |
| BMP-2 | GAGGTCCTGAGCGAGTTCGA | GGGGAAGCAGCAACACTAGA |
| GAPDH | GGAGCGAGATCCCTCCAAAAT | GGCTGTTGTCATACTTCTCATGG |

**Table S3** Primer sequences for angiogenesis-related genes

| Primer | Forward Sequence（5’-3’） | Reverse Sequence（5’-3’） |
| --- | --- | --- |
| TGF-β | GCCCTGGACACCAACTATTGCT | AGGCTCCAAATGTAGGGGCAGG |
| VEGF | CTACCTCCACCATGCCAAGT | GCAGTAGCTGCGCTGATAGA |
| CD31 | CACCAACAGCGTCAACAGTG | GCTGGTGTTGTTGGTGGTCT |
| GAPDH | GGAGCGAGATCCCTCCAAAAT | GGCTGTTGTCATACTTCTCATGG |

**Table S4** Primer sequences for inflammation-related genes

| Primer | Forward Sequence（5’-3’） | Reverse Sequence（5’-3’） |
| --- | --- | --- |
| CD206 | CTCTGTTCAGCTATTGGACGC | CGGAATTTCTGGGATTCAGCTTC |
| IL-10 | GCTCTTACTGACTGGCATGAG | CGCAGCTCTAGGAGCATGTG |
| TNF-α | CCCTCACACTCAGATCATCTTCT | GCTACGACGTGGGCTACAG |
| IL-1β | GCAACTGTTCCTGAACTCAACT | ATCTTTTGGGGTCCGTCAACT |
| GAPDH | AGGTCGGTGTGAACGGATTTG | TGTAGACCATGTAGTTGAGGTCA |

**Table S5** Adjusted P values, effect sizes, and 95% confidence intervals for pairwise comparisons of the quantitative shown in Figure 7B

| Time | Comparison | P value | Hedges’g | Mean difference (95% CI) |
| --- | --- | --- | --- | --- |
| 6W | nnGS - Control | 0.0006 | 3.813 | 10.980 (5.847–16.112) |
| 6W | nGS - Control | 0.02769 | 2.391 | 5.812 (0.679–10.945) |
| 6W | HAp - Control | 0.04139 | 2.827 | 5.349 (0.216–10.481) |
| 6W | nnGS - nGS | 0.0485 | 1.77 | 5.168 (0.035–10.300) |
| 6W | nnGS - HAp | 0.03238 | 2.262 | 5.631 (0.498–10.764) |
| 6W | nGS - HAp | 0.99092 | 0.237 | 0.464 (−4.669–5.596) |
| 12W | nnGS - Control | 0.00002 | 6.999 | 21.490 (15.204–27.776) |
| 12W | nGS - Control | 0.00028 | 4.973 | 15.002 (8.716–21.288) |
| 12W | HAp - Control | 0.03748 | 2.094 | 6.690 (0.404–12.976) |
| 12W | nnGS - nGS | 0.04327 | 2.301 | 6.488 (0.202–12.774) |
| 12W | nnGS - HAp | 0.00031 | 4.917 | 14.800 (8.514–21.086) |
| 12W | nGS - HAp | 0.0122 | 2.813 | 8.312 (2.026–14.598) |

**Table S6** Adjusted P values, effect sizes, and 95% confidence intervals for pairwise comparisons of the quantitative shown in Figure 7C

| Time | Comparison | P value | Hedges’g | Mean difference (95% CI) |
| --- | --- | --- | --- | --- |
| 6W | nnGS - Control | 0.00741 | 3.022 | 6.993 (2.146–11.841) |
| 6W | nGS - Control | 0.29277 | 1.219 | 2.905 (−1.942–7.752) |
| 6W | HAp - Control | 0.57805 | 0.911 | 1.994 (−2.853–6.841) |
| 6W | nnGS - nGS | 0.10144 | 1.676 | 4.088 (−0.759–8.935) |
| 6W | nnGS - HAp | 0.04342 | 2.222 | 4.999 (0.152–9.847) |
| 6W | nGS - HAp | 0.92863 | 0.393 | 0.911 (−3.936–5.758) |
| 12W | nnGS - Control | 0.00007 | 5.976 | 12.012 (7.834–16.189) |
| 12W | nGS - Control | 0.00161 | 4.003 | 7.703 (3.525–11.880) |
| 12W | HAp - Control | 0.4556 | 0.985 | 2.019 (−2.158–6.197) |
| 12W | nnGS - nGS | 0.0434 | 2.218 | 4.309 (0.132–8.486) |
| 12W | nnGS - HAp | 0.00027 | 4.833 | 9.992 (5.815–14.170) |
| 12W | nGS - HAp | 0.0104 | 2.864 | 5.683 (1.506–9.861) |

**Table S7** Adjusted P values, effect sizes, and 95% confidence intervals for pairwise comparisons of the quantitative shown in Figure 8B

| Time | Comparison | P value | Hedges’g | Mean difference (95% CI) |
| --- | --- | --- | --- | --- |
| 6W | nnGS - Control | <0.00001 | 16.668 | 4.355 (3.715–4.995) |
| 6W | nGS - Control | <0.00001 | 8.388 | 3.344 (2.704–3.984) |
| 6W | HAp - Control | 0.00302 | 4.5 | 1.070 (0.430–1.710) |
| 6W | nnGS - nGS | 0.00429 | 2.788 | 1.012 (0.371–1.652) |
| 6W | nnGS - HAp | <0.00001 | 19.182 | 3.285 (2.645–3.925) |
| 6W | nGS - HAp | 0.00002 | 6.564 | 2.273 (1.633–2.913) |
| 12W | nnGS - Control | <0.00001 | 15.319 | 5.564 (4.905–6.223) |
| 12W | nGS - Control | <0.00001 | 9.819 | 3.324 (2.666–3.983) |
| 12W | HAp - Control | 0.02346 | 3.288 | 0.771 (0.112–1.430) |
| 12W | nnGS - nGS | 0.00002 | 5.891 | 2.240 (1.581–2.898) |
| 12W | nnGS - HAp | <0.00001 | 16.452 | 4.793 (4.134–5.452) |
| 12W | nGS - HAp | <0.00001 | 9.823 | 2.554 (1.895–3.212) |

**Table S8** Adjusted P values, effect sizes, and 95% confidence intervals for pairwise comparisons of the quantitative shown in Figure 8C

| Time | Comparison | P value | Hedges’g | Mean difference (95% CI) |
| --- | --- | --- | --- | --- |
| 6W | nnGS - Control | <0.00001 | 13.555 | 4.945 (4.321–5.569) |
| 6W | nGS - Control | 0.00002 | 7.781 | 2.166 (1.542–2.790) |
| 6W | HAp - Control | 0.03250 | 3.311 | 0.684 (0.060–1.308) |
| 6W | nnGS - nGS | <0.00001 | 7.556 | 2.779 (2.155–3.403) |
| 6W | nnGS - HAp | <0.00001 | 13.445 | 4.261 (3.637–4.885) |
| 6W | nGS - HAp | 0.00029 | 6.998 | 1.482 (0.858–2.106) |
| 12W | nnGS - Control | <0.00001 | 14.48 | 3.995 (3.480–4.510) |
| 12W | nGS - Control | <0.00001 | 7.514 | 2.272 (1.756–2.787) |
| 12W | HAp - Control | 0.01146 | 2.619 | 0.689 (0.174–1.204) |
| 12W | nnGS - nGS | 0.00002 | 7.543 | 1.723 (1.208–2.239) |
| 12W | nnGS - HAp | <0.00001 | 19.091 | 3.306 (2.791–3.821) |
| 12W | nGS - HAp | 0.00004 | 7.438 | 1.583 (1.067–2.098) |

**Table S9** Adjusted P values, effect sizes, and 95% confidence intervals for pairwise comparisons of the quantitative shown in Figure 8D

| Time | Comparison | P value | Hedges’g | Mean difference (95% CI) |
| --- | --- | --- | --- | --- |
| 6W | nnGS - Control | <0.00001 | 22.541 | 3.581 (3.279–3.884) |
| 6W | nGS - Control | 0.00006 | 7.962 | 0.886 (0.583–1.189) |
| 6W | HAp - Control | 0.00312 | 5.944 | 0.504 (0.201–0.807) |
| 6W | nnGS - nGS | <0.00001 | 14.46 | 2.695 (2.393–2.998) |
| 6W | nnGS - HAp | <0.00001 | 17.901 | 3.077 (2.775–3.380) |
| 6W | nGS - HAp | 0.01581 | 2.957 | 0.382 (0.079–0.685) |
| 12W | nnGS - Control | <0.00001 | 13.421 | 5.864 (5.098–6.631) |
| 12W | nGS - Control | <0.00001 | 10.323 | 4.067 (3.300–4.833) |
| 12W | HAp - Control | 0.00111 | 4.271 | 1.495 (0.729–2.262) |
| 12W | nnGS - nGS | 0.00031 | 4.707 | 1.798 (1.031–2.564) |
| 12W | nnGS - HAp | <0.00001 | 12.981 | 4.369 (3.602–5.135) |
| 12W | nGS - HAp | 0.00002 | 9.233 | 2.571 (1.805–3.338) |

**Table S10** Adjusted P values, effect sizes, and 95% confidence intervals for pairwise comparisons of the quantitative shown in Figure 8E

| Time | Comparison | P value | Hedges’g | Mean difference (95% CI) |
| --- | --- | --- | --- | --- |
| 6W | nnGS - Control | 0.00004 | −6.054 | −0.881 (−1.161–−0.602) |
| 6W | nGS - Control | 0.00643 | −2.761 | −0.414 (−0.693–−0.134) |
| 6W | HAp - Control | 0.00602 | 2.259 | 0.418 (0.138–0.698) |
| 6W | nnGS - nGS | 0.00303 | −12.025 | −0.468 (−0.748–−0.188) |
| 6W | nnGS - HAp | <0.00001 | −11.248 | −1.299 (−1.579–−1.020) |
| 6W | nGS - HAp | 0.00006 | −6.886 | −0.832 (−1.111–−0.552) |
| 12W | nnGS - Control | <0.00001 | −9.584 | −0.820 (−0.970–−0.671) |
| 12W | nGS - Control | 0.00057 | −3.310 | −0.322 (−0.471–−0.172) |
| 12W | HAp - Control | 0.02384 | −1.946 | −0.174 (−0.323–−0.025) |
| 12W | nnGS - nGS | 0.00002 | −10.686 | −0.499 (−0.648–−0.350) |
| 12W | nnGS - HAp | <0.00001 | −23.794 | −0.646 (−0.796–−0.497) |
| 12W | nGS - HAp | 0.0526 | −2.762 | −0.148 (−0.297–0.002) |

**Table S11** Adjusted P values, effect sizes, and 95% confidence intervals for pairwise comparisons of the quantitative shown in Figure 8F

| Time | Comparison | P value | Hedges’g | Mean difference (95% CI) |
| --- | --- | --- | --- | --- |
| 6W | nnGS - Control | <0.00001 | 16.181 | 4.563 (4.113–5.013) |
| 6W | nGS - Control | <0.00001 | 16.756 | 2.085 (1.635–2.535) |
| 6W | HAp - Control | 0.04382 | 4.099 | 0.463 (0.013–0.913) |
| 6W | nnGS - nGS | <0.00001 | 8.773 | 2.478 (2.028–2.928) |
| 6W | nnGS - HAp | <0.00001 | 14.77 | 4.100 (3.650–4.550) |
| 6W | nGS - HAp | 0.00001 | 14.219 | 1.622 (1.172–2.072) |
| 12W | nnGS - Control | 0.0003 | 5.568 | 1.285 (0.742–1.828) |
| 12W | nGS - Control | <0.00001 | 7.723 | 2.278 (1.735–2.821) |
| 12W | HAp - Control | 0.01102 | 7.834 | 0.731 (0.188–1.274) |
| 12W | nnGS - nGS | 0.00171 | −2.788 | −0.993 (−1.535–−0.450) |
| 12W | nnGS - HAp | 0.04566 | 2.515 | 0.554 (0.011–1.097) |
| 12W | nGS - HAp | 0.00008 | 5.393 | 1.546 (1.003–2.089) |

**Table S12** Adjusted P values, effect sizes, and 95% confidence intervals for pairwise comparisons of the quantitative shown in Figure 8G

| Time | Comparison | P value | Hedges’g | Mean difference (95% CI) |
| --- | --- | --- | --- | --- |
| 6W | nnGS - Control | <0.00001 | 17.36 | 4.765 (4.323–5.208) |
| 6W | nGS - Control | 0.00002 | 27.193 | 1.555 (1.112–1.998) |
| 6W | HAp - Control | 0.03966 | 4.239 | 0.466 (0.023–0.909) |
| 6W | nnGS - nGS | <0.00001 | 11.524 | 3.210 (2.767–3.653) |
| 6W | nnGS - HAp | <0.00001 | 14.627 | 4.300 (3.857–4.743) |
| 6W | nGS - HAp | 0.00022 | 9.1 | 1.089 (0.646–1.532) |
| 12W | nnGS - Control | <0.00001 | 17.278 | 8.081 (7.299–8.863) |
| 12W | nGS - Control | <0.00001 | 17.686 | 4.435 (3.653–5.217) |
| 12W | HAp - Control | 0.01692 | 15.088 | 0.974 (0.192–1.756) |
| 12W | nnGS - nGS | <0.00001 | 6.948 | 3.646 (2.864–4.428) |
| 12W | nnGS - HAp | <0.00001 | 15.27 | 7.107 (6.325–7.889) |
| 12W | nGS - HAp | <0.00001 | 14.042 | 3.461 (2.679–4.243) |

References:

1.	Dhivya S, Saravanan S, Sastry TP, Selvamurugan N. Nanohydroxyapatite-reinforced chitosan composite hydrogel for bone tissue repair in vitro and in vivo. *J Nanobiotechnology* 2015;13:40.

2.	Zhang H, Huang H, Hao G, Zhang Y, Ding H, Fan Z, Sun L. 3D Printing Hydrogel Scaffolds with Nanohydroxyapatite Gradient to Effectively Repair Osteochondral Defects in Rats. *Advanced Functional Materials* 2020.

3.	Zhang X, Wang X, Qiu H, Kong D, Han M, Guo Y. Poly(methacrylate citric acid) with good biosafety as nanoadsorbents of heavy metal ions. *Colloids Surf B Biointerfaces* 2020;187:110656.

4.	Qi Q, Chu M, Yu X, Xie Y, Li Y, Du Y, Liu X, Zhang Z, Shi J, Yan N. Anthocyanins and Proanthocyanidins: Chemical Structures, Food Sources, Bioactivities, and Product Development. *Food Reviews International* 2023;39:30.

5.	Tao W, Zhang G, Liu C, Jin L, Li X, Yang S. Low-dose LPS alleviates early brain injury after SAH by modulating microglial M1/M2 polarization via USP19/FOXO1/IL-10/IL-10R1 signaling. *Redox Biol* 2023;66:102863.
